# Establishing a Developmentally Appropriate fMRI Paradigm Relevant to Presurgical Mapping of Memory in Children

**DOI:** 10.1007/s10548-019-00751-7

**Published:** 2019-12-21

**Authors:** Amanda G. Wood, Elaine Foley, Parnpreet Virk, Helen Ruddock, Paras Joshee, Kelly Murphy, Stefano Seri

**Affiliations:** 1 School of Life and Health Sciences, Aston Brain Centre, Aston University, Aston Triangle, Birmingham, B4 7ET UK; 2 School of Psychology, Faculty of Health, Deakin University, Burwood Campus, Deakin, VIC Australia; 3 School of Psychology, University of Birmingham, Birmingham, UK; 4 Children’s Epilepsy Surgery Service, Birmingham Women’s and Children’s Hospital, Birmingham, UK

**Keywords:** Memory, Children, fMRI, Epilepsy, Lateralisation, Asymmetry

## Abstract

Functional magnetic resonance imaging (fMRI) is an established eloquent cortex mapping technique that is now an integral part of the pre-operative work-up in candidates for epilepsy surgery. Emerging evidence in adults with epilepsy suggests that material-specific fMRI paradigms can predict postoperative memory outcomes, however these paradigms are not suitable for children. In pediatric age, the use of memory fMRI paradigms designed for adults is complicated by the effect of developmental stages in cognitive maturation, the impairment experienced by some people with temporal lobe epilepsy (TLE) and the normal representation of memory function during development, which may differ from adults. We present a memory fMRI paradigm designed to activate mesial temporal lobe structures that is brief, independent of reading ability, and therefore a novel candidate for use in children. Data from 33 adults and 19 children (all healthy controls) show that the paradigm captures the expected leftward asymmetry of mesial temporal activation in adults. A more symmetrical pattern was observed in children, consistent with the progressive emergence of hemispheric specialisation across childhood. These data have important implications for the interpretation of presurgical memory fMRI in the pediatric setting. They also highlight the need to carefully consider the impact of cognitive development on fMRI tools used in clinical practice.

## Introduction

There is strong evidence that functional magnetic resonance imaging (fMRI) is a clinically suitable alternative to more invasive procedures that establish lateralization of cognitive function. Although the evidence is stronger for language mapping, there is emerging evidence that preoperative asymmetry in verbally-mediated tasks provides information about risk to postoperative verbal memory (Szaflarski et al. 2017), consistent with traditional material-specific accounts of verbal memory risk in left temporal lobe epilepsy surgery (Saling 2009).

Predicting cognitive outcome following surgery to eloquent cortex in children, however, cannot rely on current guidance, which drew on studies in adults. A number of methodological factors play an important role, however the single most important issue that remains unresolved in the literature is the normal hemispheric representation of function in the developing brain and the role of this in interpreting patterns of activation in preoperative fMRI studies.

The usual patterns of language lateralization expected in healthy controls or people with epilepsy undergoing evaluation for surgery can be demonstrated with fMRI language paradigms. Although there is variability in task design and analytic approach (Benjamin et al. 2018), most studies show good overall concordance between fMRI and the gold-standard intracarotid sodium amytal procedure (IAP, or “Wada” test; Dym et al. 2011). In adults, the usual proportion of left-lateralized language representation is observed, with variation associated with left handedness (Berl et al. 2005). In people with epilepsy, the proportion with right-sided or bilateral activation/representation increases in those assessed for resective surgery, particularly those with temporal lobe epilepsy. Again, the association between atypical language representation and handedness is observed (Berl et al. 2005).

Functional imaging of language is successful in children aged six years or more (Wood et al. 2004) but unlike adults, younger children show bilateral activation, with the typical left-lateralised patterns seen only later in childhood/adolescence (Szaflarski et al. 2006). Thus, depending on the age or developmental stage of the child, it is usual to see some degree of contralateral activation in a normally well-lateralised task. Interpretation of bilateral activation is therefore complicated in children with TLE and necessitates a clear discrimination between the presence of bilateral activation that represents truly atypical language dominance from normal developmental representation of emerging cognitive skills (Gonzalez et al. 2014).

The representation of language is relevant to the measurement of hemispheric memory functions. Although more recent studies call in to question the veracity of the traditional material-specific model of memory function (Saling 2009), there is a strong association between hemispheric representation of language and other verbally-mediated tasks particularly in adults. Indeed, although there are case reports describing differential hemispheric representation of language and verbal memory (Wood et al. 1999) the overwhelming clinical presentation in adults is of *co-lateralization* of functions. This raises the possibility that interpretation of fMRI memory data in children should consider the left-right variation across development of language representation. However, evidence for a so-called material-specific memory profile in children (e.g. Jambaque 2008; Schoenfeld et al. 1999) is mixed, with several studies failing to uncover material-specific cognitive deficits in children (Baxendale et al. 2010; Gonzalez et al. 2007; Lendt et al. 1999; Smith et al. 2007). This reflects findings in typically developing populations, in whom there is an association between increased hippocampal volume and and episodic memory scores (Lee et al. 2014), suggesting that as memory skills are attained there is concomitant brain development. This extends to functional network connections, with modified interaction between left mesial temporal and left prefrontal cortex across childhood during scene encoding paradigms (Menon et al. 2005). Data also support an increasing role of the left mesial temporal lobe in recognition memory performance as children develop, left anterior medial temporal lobe and left prefrontal cortex activation predicting subsequent memory older but not younger children (Chiu et al. 2006). Whilst these data highlight the increasing interplay of the prefrontal cortex with the medial temporal lobe to support memory performance in later stages of children’s cognitive development, they also support the clinical literature which points to an increasing role for the left mesial temporal lobe in verbal encoding, at least, across childhood. Thus, paradigms designed for paediatric cohorts within a clinical setting are warranted.

Memory fMRI is used successfully in adults with temporal lobe epilepsy and shows some promise for predicting postoperative memory outcome. The majority of tasks used show bilateral activation in the mesial temporal lobe. There are a number of commonly used paradigms (Towgood et al. 2015) that feature in studies whose aim is to predict postoperative memory status. *Hometown Walking* requires participants to imagine a route that is personal/familiar to them during scanning and this active phase is compared to a control condition of counting. *Visual Scenes* presents participants with complex images that are then compared to scrambled versions of the same. *Words and Pictures* is a modified version from Powell to exclude face encoding. Instead it presents blocks of concrete nouns or blocks of colour pictures, which are compared to a baseline of cross-hair fixation. In the latter two tasks, participants are instructed to memorise the items for later recognition and each item required a like/dislike judgment to encourage ‘deep encoding’ (Nyberg 2002). In a recent study of 16 adults with temporal lobe epilepsy, all three activation paradigms showed bilateral mesial temporal activation (Towgood et al. 2015). Analysis of word encoding using an event-related, rather than block design, approach proved most reliable of all the tasks, when taking in to account between-sessions variability in activation, left-right mesial temporal activation and ability to classify the laterality of patients’ seizure foci (Towgood et al. 2015).

Nevertheless, tasks that demand reading words are not suitable for young children whose reading skills are not expected to be as well developed as their older peers or adults and so aural presentation of words is preferable. The instruction to remember items for later recall may also encourage different encoding and consolidation strategies in adults and young children (Bjorklund and de Marchena 1984), which in turn may affect patterns of activation and resultant interpretation of group differences. Thus, an *incidental* rather than intentional encoding paradigm is better suited for studies involving children. The feasibility of these methodological changes is yet to be determined in a pediatric setting.

The desire to predict risk of postoperative memory decline requires careful consideration of factors likely to influence patterns of activation. The right mesial temporal activation observed in adults during verbal memory tasks (Richardson et al. 2003) has been interpreted as evidence of reorganization of function to the contralesional temporal lobe. Consistent with the neuropsychological indices of hippocampal integrity and postoperative decline (Helmstaedter and Kockelmann 2006) left mesial temporal activation is a strong predictor of outcome, with greater activation inversely correlated with postoperative verbal memory (Richardson et al. 2006). Given that language and verbal memory lateralization typically co-occur (Wood et al. 1999), mesial temporal activation during verbal encoding may occur bilaterally in children, even in the absence of left mesial temporal pathology. Consistent with this, a recent study using a language paradigm to assess lateralization in MTL in children and adults found that adults were more left lateralized than children (Sepeta et al. 2016). Thus, unilateral or asymmetric mesial temporal activation may not be a suitable marker of post-operative memory outcomes in children.

The primary aim of this study was to evaluate the pattern of activation in children during a developmentally appropriate verbal encoding paradigm. In doing so, we also aimed to determine whether the lateral representation of verbal memory activation differed in young children compared to older participants. We predicted that young children would exhibit bilateral mesial temporal activation during the task compared to adults, who would show typical asymmetric activation.

## Methods

### Participants

We recruited adults (18 years or more) and children (17 years of age or younger) without a history of neurological diagnosis through community advertisements and word of mouth. In this report, we present data from right-handed participants only. Thirty-six adults (15 male, age range 18–44, mean = 22.9 years, SD 5.9 years) and 19 children (10 male, age range 8–16 years, mean = 11.8 years, SD 2.6) successfully completed the scanning procedure. Three adults recruited to the study were removed from the current analyses; based on their age at assessment they were statistical outliers (± 2SD greater than the group mean) and so data are presented in that group on 33 adults (13 male, age range 18–30, mean = 21.3, SD 2.7).

Adults and parents/guardians of minors provided written informed consent for their child and all children assented to the study. The ethics committees of Aston University (#888) and University of Birmingham (ERN_11-0429), UK, approved the work.

### Verbal Memory Paradigm

The fMRI paradigm comprised an incidental verbal encoding task presented binaurally. The overall study design involved three phases: (1) out of scanner familiarization, (2) in-scanner encoding, (3) out of scanner recognition. Words were selected on the basis that they contained 1 to 2 syllables and had an age of acquisition (AoA) rating of less than 600. The latter was derived from an open resource (http://websites.psychology.uwa.edu.au/school/MRCDatabase/mrc2.html) that multiplied the rating scale of Gilhooly and Logie (1980) by 100. Half of the words selected were ‘living’ and half were ‘non-living’ and these were balanced for AoA, familiarity, imageability, and length. During familiarization, participants listened to two words presented binaurally alternately, five times via headphones. Participants pressed a button to indicate whether words were living or non-living to ensure that rehearsal strategies were not employed differentially in adults and children and encourage deep encoding via semantic access. This established them as the ‘familiar’ words for the subsequent ‘novel’ versus ‘familiar’ contrast, because there is strong evidence of enhanced hippocampal activation to novelty (Gabrieli et al. 1997; Stern et al. 1996).

During scanning, words were presented in alternating blocks of ‘novel’ and ‘familiar’ (i.e. repeated) items (5 items per block; 4 blocks novel, 5 blocks familiar), with participants responding ‘living’ or ‘non-living’ via button-press. To overcome scanner noise, words were presented via headphones during the TR delay. To minimize head movement, participants were instructed to focus on a black cross hair presented in the center of a back-projected screen which was located at the rear of the scanner. To reduce the time of scanning, two separate runs were performed using different novel words and these were counterbalanced across participants. The total scanning time per memory run was approximately three minutes. Two runs were completed.

Following scanning and in a room within the imaging suite but outside the scanner, a surprise post-recognition memory test (‘old’ fMRI words versus ‘new’ foils) determined which items were successfully encoded, for later analysis. This was achieved by asking participants to listen to words on a laptop and indicate via button press whether the word was ‘old’ (ie they heard it during when they were inside the scanner) or ‘new’ (no, they did not hear it before now). Only correctly recognized words were included in further analyses by creating custom timing files for each participant to indicate which were correctly encoded.

### Image Acquisition

Data were acquired at two centers, Birmingham University Imaging Centre (BUIC; Philips Achieva 3 T MRI) and Aston Brain Centre (ABC; Siemens Trio Tim, 3 T system), Birmingham, United Kingdom. Whole brain, high-resolution T1-weighted images were acquired for image co-registration in the sagittal plane at both sites (BUIC parameters: TR 8.4 s; TE 3.8 s; voxel size 1 mm isotropic; flip angle 8°; 175 slices. ABC parameters: TR 1.9 s; TE 3.4 s; voxel size 1 mm isotropic; flip angle 15°; 176 slices).

Functional MRI data were acquired with an echo-planar acquisition protocol to measure the blood-oxygen level dependent (BOLD) response (ABC: TR 4110 ms, delay 2300 ms; TE 30 ms; 2 mm isotropic voxels; flip angle 90°; 26 slices. BUIC: TR 4000 ms, delay 2300 ms; TE 35 ms, 2.3 × 2.3 × 2.5 mm voxels; flip angle 80°; 26 slices). To maximize sensitivity to mesial temporal structures data were acquired in a restricted region with the field of view covering the hippocampus and neighboring temporal lobe. On sagittal views the anterior–posterior axis was aligned with the long axis of the hippocampus and the body of the hippocampus was in the center.

### Data Analysis

#### Data Preprocessing

All data were analyzed using statistic parametric mapping (SPM12) running in Matlab R2012a (http://www.fil.ion.ucl.ac.uk/spm/). Dummy scans were included prior to the first trial acquisition thus no volumes were removed from the series prior to analysis. Slice time correction was applied, followed by realignment where the mean image was used as reference. 6 motion parameters were extracted during realignment and used as regressors in the whole-brain analysis. A pre-established exclusion criterion was movement exceeding 2 mm (~ 1 voxel size) total displacement. The functional data were initially co-registered to individual anatomical images and then transformed to the standard SPM12 MNI EPI template to facilitate group comparisons. Finally, a 6 mm^3^ full width by half maximum (FWHM) isotropic gaussian kernel was used to spatially smooth the normalized images.

#### Whole-Brain Analyses

Statistical analysis at the individual level was estimated using the general linear model (GLM) in SPM12. The first model regressor was input as the novel condition and second regressor as the familiar condition. Movement regressors obtained during preprocessing were included in the model to control for variance associated with in-scanner movement. A high pass filter of 128 s cut-off was implemented to remove low frequency noise components. The key contrast was between encoded novel versus familiar words for whole-brain analyses. Group analysis was performed using a random-effects model and scanner site was included as covariate.

#### Region of Interest Analyses

Regions of interest (ROIs) were defined for each participant using the automatic anatomic labels (Tzourio-Mazoyer et al. 2002) in the Wake Forest Pick Atlas (Maldjian et al. 2003, 2004). Two ROIs were created for each participant; bilateral mesial temporal masks (MTL; including hippocampi and parahippocampal gyri) and bilateral superior temporal masks (STG; including middle and superior temporal gyri).

Memory asymmetry indices were estimated for the MTL and STG ROIs at individual and group level using the LI Toolbox, where threshold-independent lateralization indices were computed using a bootstrap method (Wilke and Lidzba 2007). Lateralization was categorized as left, right, or bilateral based on a commonly used value of 0.20. ROIs were categorized individually as left lateralized if LI ≥ 0.20, bilateral if LI < │0.20│, or right if LI ≤ − 0.20.

Analysis of variance (ANOVA) was used to assess group differences in MTL and STG laterality between adults and children to provide information on the average LI scores. Chi-square analysis was used to assess whether the proportion of left/right/bilateral LI scores differed between the groups. Given that the resultant sample of children and adults included cases at the age boundary (i.e. 16 for children and 18 for adults), we also exampled the relationship between age and LI scores using Pearson’s correlation across the whole sample. Only individuals with activation clusters of at least 5 voxels were included in the LI analyses (Wilke and Lidzba 2007).

## Results

All participants completed the task with an average correct recognition rate of 80% (SD 10.2) for adults and 82% (SD 9.7) for children. Only those words that were correctly recognised during the memory recall task were used in the fMRI analysis of the verbal encoding paradigm.

**Fig. 1:**
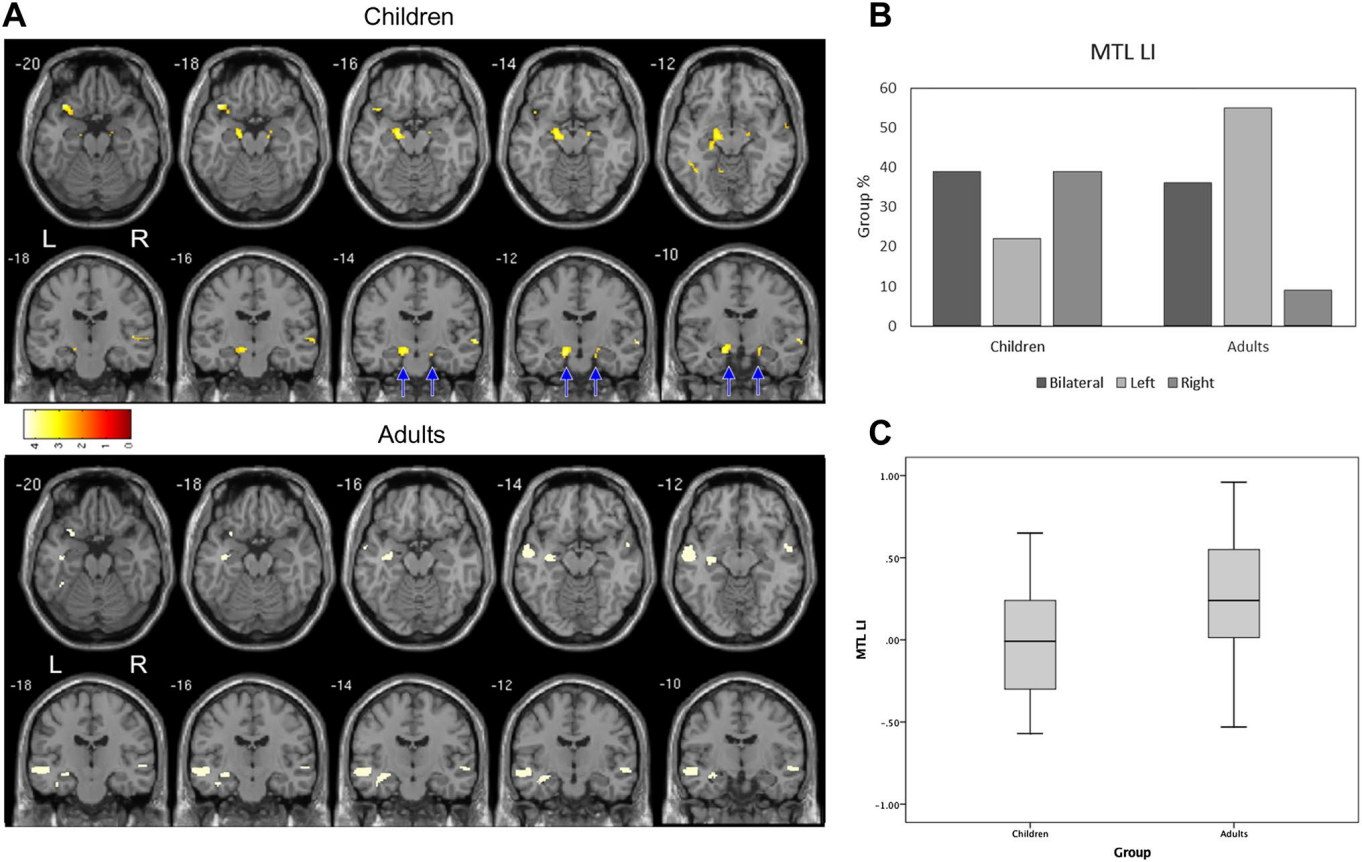
**a** Group level results for correctly remembered novel words during a verbal encoding task. Significant group level activation in children (n = 19) and adults (n = 33), both p < .05 corrected. **b** Proportion of the groups with left/right/bilateral activation during verbal encoding. **c** Significant group difference in average LI scores

We first established group level MTL activation, to demonstrate the paradigm’s pattern of activation. At the group level adults showed the expected pattern of leftward activation within the mesial temporal lobe (p < 0.001 corrected, T = 3.38; Fig. 1a). Conversely, at the group level, the child cohort showed evidence of bilateral activation (see Fig. 1a). There was a significant difference in MTL LI between adults and children F(1,51) = 7.49, p = 0.009 (Fig. 1a). This group difference was not observed in the STG ROI.

**Fig. 2:**
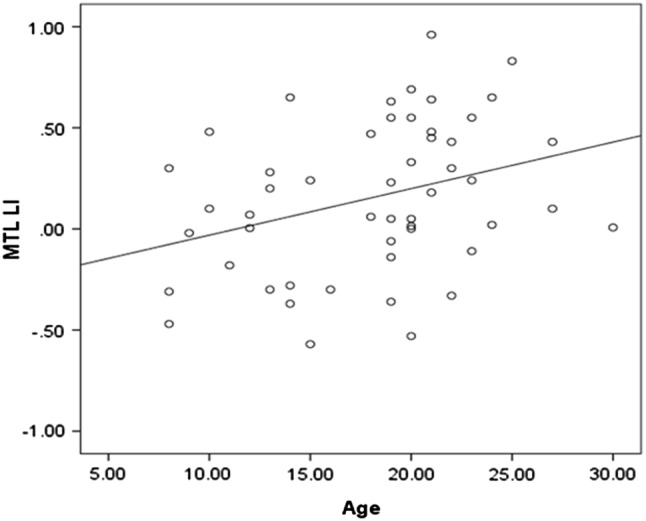
Scatterplot showing a significant correlation (r = 0.33) between age and memory asymmetry index (LI) in adults and children in the left mesial temporal region of interest

Average LI scores preclude an understanding of clinically relevant lateralization information and so we next examined individual level data. At the individual level, all of the adults (n = 33) and 18 of 19 children demonstrated individual MTL activation with clusters of at least 5 voxels (see Fig. 1c). Overall 55% of adults were identified as left lateralized in MTL, 36% were bilateral and 9% were right lateralized. In contrast 22% of children were categorized as left lateralized in MTL, 39% were bilateral and 39% were right lateralized. There was a significant difference in the proportion of left/right/bilateral LI scores for the MTL in the two groups (χ^2^(2) = 8.2, p = 0.016; Fig. 1b). Given that the two groups that were recruited to the study included participants with similar ages (i.e. children aged 16 and adults aged 18) we examined the development of lateralisastion by examining the association between age and LI score. In the MTL ROI there was a significant relationship between age and LI (r = 0.33, p = 0.019; Fig. 2), whereas no such relationship was observed for the STG ROI (r = 0.04, p = 0.79)

## Discussion

Our study showed that a brief, developmentally-appropriate paradigm can be used to map activation during verbal encoding, activating MTL structures at individual and group level in a cohort of healthy adults and children. The task yields more leftward asymmetry in healthy right-handed adults and, as predicted, the pattern of activation observed in children differed from adults, demonstrating more frequent right lateralised MTL activation in children. The task was chosen with a view to assess feasibility and to acquire normative data for a later use in the context of pre-surgical evaluation in young patients with drug resistant epilepsy, where resection of the left temporal lobe is associated with risk of verbal memory impairment. Children tolerated the paradigm and broader scanning session well and no data were removed due to excessive artifact (e.g. movement). Our data support the contention that atypical MTL hemispheric lateralization of activation is a typical feature during childhood and this has significant implications for the interpretation of fMRI memory studies in the clinical setting.

We have demonstrated that a developmentally appropriate verbal encoding paradigm can be used to elicit activation in MTL in children. Of particular relevance for clinical applications is the detectability of the response at the individual level. We have demonstrated that memory asymmetry can be detected at the individual level in children using a threshold-independent bootstrap technique. The importance of threshold-independent methods for calculating lateralization/asymmetry indices has been acknowledged (Bradshaw et al. 2017), with the aim to increase standardization and reliability of interpretation across fMRI laterality protocols in general and particularly in a clinical setting.

The ultimate goal of fMRI memory studies during presurgical evaluation is to predict postoperative neuropsychological deficits, where the prediction of verbal memory decline in individual patients is most relevant. Recent AAN Practice Guidelines indicate moderate evidence for memory fMRI paradigms predicting post-surgical outcome in adults (Szaflarski et al. 2017). However, very few studies have focused on determining verbal memory lateralization in children. This not only requires a reliable age-appropriate task to elicit activation within the desired network but also understanding of the underlying representation and developmental changes. The majority of previous fMRI memory studies in typically developing children have focused on visual memory (for review see Sepeta et al. 2018), where age-related differences in hippocampal activation have been reported (Demaster and Ghetti 2013). There is evidence to suggest that the functional changes that occur during development result in the hippocampus and parahippocampal gyrus becoming increasingly more specialised for detailed recollection (Ghetti et al. 2010). Thus, our data highlight the importance of understanding the typical patterns of activation seen across childhood in order to interpret data in pediatric clinical cohorts.

The development of leftward asymmetry during verbally mediated tasks occurs for traditional language processing tasks in not only expected cortical regions (e.g. IFG and STG) but also within the mesial temporal lobes (Binder et al. 2008; Sepeta et al. 2016). During a language task, Sepeta et al. (2016) found a developmental difference in MTL lateralization, where MTL activation was left lateralized for adults, but more bilateral in children. In addition, they found that language lateralization indices within traditional language regions (i.e. Broca’s and Wernicke’s) were associated with MTL lateralization for adults, but not for children. These data support the idea that either language tasks or relevant memory tasks per se (e.g. declarative memory encoding) could be used to predict memory outcome post-operatively in adults (Binder et al. 2008; Szaflarski et al. 2017) but less so in children, due to the variable distribution of MTL lateralization which is unrelated to language lateralization. However, this appears to apply best in cases of typical language representation where the concordance between language and mnemonic skills appears to be strong, but there is less concordance in cases of atypical language representation (Sepeta et al. 2016). Accordingly, it might be necessary to use fMRI tasks that assay specific cognitive processes if a sensitive marker of atypical representation is needed, as is the case in clinical decision-making prior to surgery in the MTL where memory functions are at risk. Comparing the utility of different paradigms in such cases using paradigms such as the one we present here is important focus of future research; it is precisely these cases for whom advanced imaging offers the potential to avoid more invasive – yet more sensitive and specific – tools to determine lateralization of eloquent cortex.

Many studies cite strong concordance between fMRI and IAP procedures (particularly for language representation). However, the overall group rates ignore the relatively poor performance of fMRI against IAP in atypical language cases (i.e. right or, particularly, bilateral), a point acknowledged in recent practice guidelines (“The imperfect concordance between fMRI and IAP language lateralization leaves open the question of which test is more accurate in discordant cases.“ p. 399; Szaflarski et al. 2017). The data from our adult group revealed a relatively large proportion (36%) with bilateral verbal memory representation. Previous fMRI memory studies in healthy adults have also reported bilateral responses in MTL during various memory paradigms (Towgood et al. 2015). Thus, bilateral fMRI activation in adults cannot be assumed to reflect true bilateral representation of function that, from a neuropsychological perspective, would act as a marker of potential cognitive reserve. The implications of this uncertainty in the adult preoperative setting are further magnified for children in whom we *expect* there to be a degree of bilateral or rightward activation. The presence of bilateral activation in young children cannot be interpreted as functional reserve of the contralateral temporal lobe. This suggests that in young children, at least, more invasive procedures may, unfortunately, be warranted. Hence, there is an urgent need to better understand the relationship of pre-operative activation and post-operative memory outcomes using the paradigm we present here. Furthermore, the presence of high rates of bilateral LI scores for MTL activation in both groups may reflect different underlying neural representation. The left-right asymmetry of activations per se does not yield insight into the ability of either MTL to subserve function post-operatively. In adults, it is assumed that activation patterns are static; presence or indeed absence of activation is assumed to reflect some marker of functional integrity that is invariant over time under normal circumstances. Conversely, ongoing skill acquisition and development necessarily links to repatterning of neural representations in children. Hence, only longitudinal studies in young cohorts can answer the question of the functional significance of atypical activation (i.e. non leftward) during a verbal encoding task. This is an important topic warranting study if we are to reach the goal of translating imaging data into clinical practice.

The relatively small numbers of younger children across the entire pediatric cohort limits the current study. It is not possible to identify the age at which the asymmetric representation of verbal encoding becomes adult-like on the basis of these data, in part because of the preponderance of adolescents in the sample. It will be important to extend our work by performing the fMRI task in larger groups of young children so that this can be clarified. Future research should also employ a longitudinal design not only to establish change over development within child participants (as mentioned above), but also to demonstrate reliability of the paradigm over time. Data in adults highlight the variability of memory-related activation (Towgood et al. 2015) and the ongoing cognitive development of children could make this a significant problem for clinically-reliable memory fMRI. For example, we do not know whether or how typical developmental trajectories influence the stability of fMRI activation in the MTL.

The BOLD signal is known to change over time in a regionally specific manner (Szaflarski et al. 2006). This is assumed to reflect changes in task-specific cognitive processing that is detected by BOLD acquisitions. However, other confounding factors may underlie these developmental changes. For example, changes in underlying vasculature and associated neurovascular coupling may contribute to BOLD signal change. This developmental change might occur independently to cognitive development per se; acquiring measures of perfusion in children would enable careful control of confounding data so that we can better understand the basis for variability in BOLD activation over time.

In order to maximize sensitivity and coverage of the hippocampus and MTL we used a limited field of view with thin slices to focus on the temporal lobes, which is consistent with previous studies in adults (Bonelli et al. 2010; Towgood et al. 2015). This acquisition protocol also reduces scan-time, which is particularly important when scanning children and patients. However this acquisition precludes whole-brain analyses and cannot be used to assess changes in brain areas other than the temporal lobes. This approach was adopted because of our desire to address the clinically driven need for a paradigm that can be used in the pediatric setting. However, this might be seen as a limitation to broader research questions and these could be readily addressed in future studies by adapting the paradigm to a whole-brain acquisition.

Recent guidelines from AAN report significant correlations between fMRI memory activation asymmetry in the hippocampus and IAP scores (Szaflarski et al. 2017), and an association between activated hippocampal voxels and scores after left IAP. These data were provided as the basis for guidance that fMRI memory may be used to replace IAP for presurgical lateralization. At present, however it is not clear that “… the published results apply to children and adolescents…” (p. 399). The paradigm presented here is well-suited to use in children, however its clinical utility in predicting memory outcomes in children being considered for temporal lobectomies needs to be studied in a larger clinical trial. A number of centres plan to collaborate to increase the speed with which this promising finding can be translated in to clinical practice.
